# Bis(1-dodecyl-4-aza-1-azoniabi­cyclo­[2.2.2]octane)tetra­iso­thio­cyanato­cobalt(II)

**DOI:** 10.1107/S2414314620000231

**Published:** 2020-01-28

**Authors:** Niels Ole Giltzau, Martin Köckerling

**Affiliations:** a Universität Rostock, Institut für Chemie, Anorganische Festkörperchemie, Albert-Einstein-Str. 3a, D-18059 Rostock, Germany; b Department Life, Light and Matter, Universität Rostock, 18051 Rostock, Germany; Vienna University of Technology, Austria

**Keywords:** cobalt(II) complex, crystal structure, Iso­thio­cyanate, DABCO

## Abstract

The title compound, [Co(C_18_H_37_N_2_)_2_(NCS)_4_], consists of a cobalt(II) ion positioned on the origin of the triclinic unit cell. It is coordinated by the N atoms of two *trans-*oriented 1-dodecyl-4-aza-1-azoniabi­cyclo­[2.2.2]octane (DABCO^+^) cations, which carry *n*-dodecyl chains at the non-coordinating N atoms. The distorted octa­hedral coordination environment of the Co^II^ ion is completed through four N atoms of iso­thio­cyanate ions, which are arranged within the equatorial plane. Non-classical hydrogen bonding of the types C—H⋯N and C—H⋯S between the filamentous mol­ecules lead to the formation of layers parallel to (001).

## Abstract 2

The title compound comprises a cobalt(II) atom, which is six coordinate by N donor atoms, four from iso­thio­cyanato ligands and two from 1-dodecyl-4-aza-1-azoniabi­cyclo­[2.2.2]octane cations. The coordination sphere is elongated octa­hedral.

## Graphical abstract

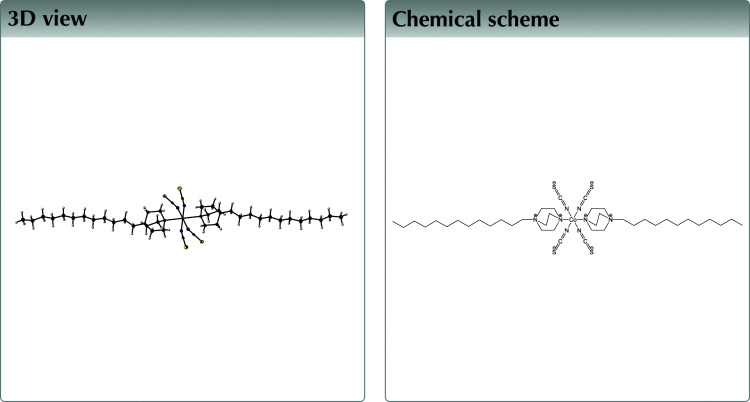

## Structure description

Ionic liquids (IL) are known as designer solvents for their special applications and properties (Santos *et al.*, 2014 ▸; Clark *et al.*, 2016 ▸), and such systems have been widely investigated over the past few years. The title compound has a low melting point and can be considered as a magnetic IL in the molten state.

**Figure 1:**
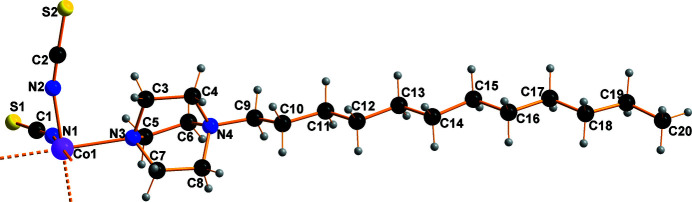
The asymmetric unit of the title compound with atom labelling.

**Figure 2:**
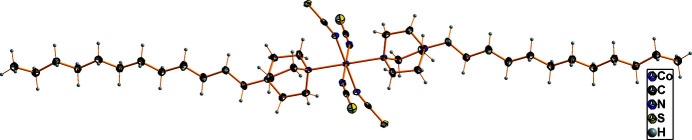
Structure of the centrosymmetric, neutral, filamentous complex mol­ecule of the title compound with the atoms being presented as 50% displacement ellipsoids.

The asymmetric unit consists of a Co_0.5_(NCS)_2_ moiety and one 1-dodecyl-4-aza-1-azoniabi­cyclo­[2.2.2]octane cation (Fig. 1 ▸), with the cobalt(II) atom located on the origin of the unit cell. Four iso­thio­cyanate groups are arranged in a twisted square plane around the Co^II^ ion. Corresponding N—Co—N bond angles are 88.95 (7)° for N1—Co1—N2(−*x*, 2 − *y*, −*z*) and 91.05 (7)° for N1—Co1—N2. The N1—C1 distance measures 1.162 (3), Å indicating a strong π-inter­action, and the C1—S1 distance is 1.629 (2) Å. The coordination polyhedron around the Co^II^ ion consists of the four N atoms of the NCS groups and two further N atoms of the positively charged DABCO ligands, leading to filamentous mol­ecules (Fig. 2 ▸). The Co—N1 and Co—N2 distances are, at 2.072 (2) and 2.090 (2) Å, in the expected range for a six-coordinate Co^II^ atom (Orpen *et al.*, 1989 ▸). With the Co—N_(DABCO)_ distances of 2.350 (2) Å, the octa­hedron is considerably elongated. This can be explained through the steric demand of the DABCO^+^ units.

**Figure 3:**
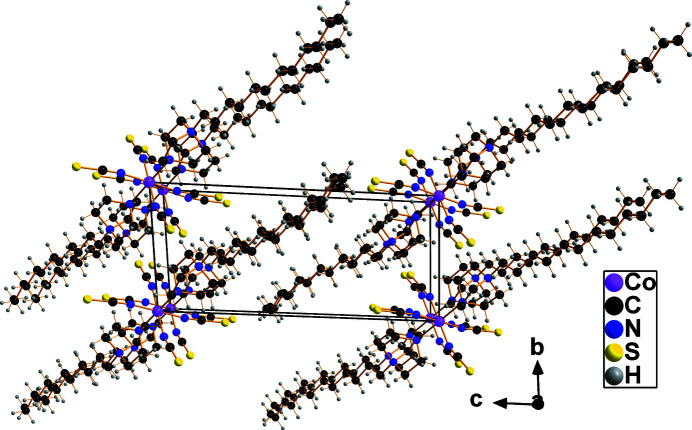
Stacking of the filamentous mol­ecules in the crystal. Hydrogen-bonding inter­actions are omitted for clarity.

In the crystal, the filamentous mol­ecules are stacked with the long *n*-dodecyl chains aligned parallel to each other (Fig. 3 ▸). Because the complex mol­ecule has no acidic H atoms, only weak, non-classical hydrogen bonds are present. Those with N as acceptor atoms are intra- and inter­molecular, those with S atoms as acceptors bridge between the filamentous mol­ecules, leading to a layer-like arrangement parallel to (001). Hydrogen-bonding parameters up to a H⋯*A* distance of 3.0 Å are listed in Table 1 ▸.

Examples of CoN_6_ coordination with four iso­thio­cyanato ligands can be found in, for example, Adach & Daszkiewicz (2016 ▸) and Wang *et al.* (2018 ▸). 1,4-Di­aza­bicylco[2.2.2]octane (DABCO) is a standard chemical in organic synthesis and catalysis, and overviews of its chemistry can be found in Baghernejad (2010 ▸), Banerjee (2018 ▸) and Yang *et al.* (2007 ▸).

## Synthesis and crystallization

The compound is accessible through the reaction of 1-dodecyl-4-aza-1-azoniabi­cyclo­[2.2.2]octane chloride with K_2_[(Co(NCS)_4_]. 1-Dodecyl-4-aza-1-azoniabi­cyclo­[2.2.2]octane chloride (Dodeca-DABCO-Cl) was prepared by the reaction of DABCO (1.4 g, 12.5 mmol) with 1-chloro­dodecane (3.6 g, 12.5 mmol) in 20 ml of aceto­nitrile. The mixture was refluxed for 10 h and the solvent removed under reduced pressure. Potassium tetra-(iso­thio­cyanato)­cobaltate(II) was prepared through the reaction of potassium iso­thio­cyanate (15 g, 154.0 mmol) with cobalt(II) chloride (5.0 g, 38.5 mmol) in 250 ml of acetone. The mixture was refluxed for 2 h, the solvent removed and the raw product extracted with ethyl acetate in a soxhlet extractor. Dodeca-DABCO-Cl (0.374 g, 1.18 mmol) and K_2_[(Co(NCS)_4_] (0.218 g, 0.59 mmol) were mixed in 10 ml of aceto­nitrile and stirred for 1 d at ambient temperature. The mixture was filtered and the solvent removed under reduced pressure. The resulting blue solid was washed several times with acetone. Large blue crystals were grown by leaving the flask open and allowing the solvent aceto­nitrile to evaporate over the course of three days. The melting point is 331 K.

## Refinement

Crystal data, data collection and structure refinement details are summarized in Table 2 ▸. Some low-angle reflections were omitted from the structure refinement because their intensities were affected by the beam stop (001, 002, 411, 311, 010, 323).

## Supplementary Material

Crystal structure: contains datablock(s) I. DOI: 10.1107/S2414314620000231/wm4120sup1.cif

Structure factors: contains datablock(s) I. DOI: 10.1107/S2414314620000231/wm4120Isup2.hkl

CCDC reference: 1976544

Additional supporting information:  crystallographic information; 3D view; checkCIF report

## Figures and Tables

**Table 1:** Hydrogen-bond geometry (Å, °)

*D*—H⋯*A*	*D*—H	H⋯*A*	*D*⋯*A*	*D*—H⋯*A*
C5—H5*A*⋯N1	0.99	2.48	3.060 (3)	117
C5—H5*A*⋯S1^i^	0.99	2.70	3.467 (2)	134
C7—H7*A*⋯S1^ii^	0.99	3.00	3.755 (2)	134
C7—H7*A*⋯N2^iii^	0.99	2.58	3.244 (3)	124
C8—H8*B*⋯S1^ii^	0.99	2.89	3.695 (2)	139
C9—H9*B*⋯S1^iv^	0.99	2.78	3.580 (2)	138

Symmetry codes: (i) 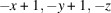 ; (ii) 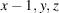 ; (iii) 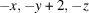 ; (iv) 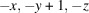 .

**Table 2:** Experimental details

Crystal data	
Chemical formula	[Co(C_18_H_37_N_2_)_2_(NCS)_4_]
*M*_r_	854.24
Crystal system, space group	Triclinic, *P* 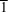
Temperature (K)	123
*a*, *b*, *c* (Å)	7.484 (1), 8.587 (1), 18.523 (2)
α, β, γ (°)	83.782 (4), 81.868 (4), 71.189 (4)
*V* (Å^3^)	1112.9 (2)
*Z*	1
Radiation type	Mo *K*α
μ (mm^−1^)	0.61
Crystal size (mm)	0.17 × 0.10 × 0.05

Data collection	
Diffractometer	Bruker Kappa APEXII CCD
Absorption correction	Multi-scan (*SADABS*; Bruker, 2017 ▸)
No. of measured, independent and observed [*I* > 2σ(*I*)] reflections	35032, 6802, 4509
*R*_int_	0.084
(sin θ/λ)_max_ (Å^−1^)	0.715

Refinement	
*R*[*F* ^2^ > 2σ(*F* ^2^)], *wR*(*F* ^2^), *S*	0.049, 0.106, 1.04
No. of reflections	6802
No. of parameters	241
H-atom treatment	H-atom parameters constrained
Δρ_max_, Δρ_min_ (e Å^−3^)	0.53, −0.65

Computer programs: *APEX2* and *SAINT* (Bruker, 2017 ▸), *SHELXT* (Sheldrick, 2015*a* ▸), *SHELXS* (Sheldrick, 2015*b* ▸), *DIAMOND* (Brandenburg & Putz, 2019 ▸) and *publCIF* (Westrip, 2010 ▸).

## full crystallographic data

### Crystal data

**Table:**

[Co(C_18_H_37_N_2_)_2_(NCS)_4_]	*F*(000) = 461
*M**_r_* = 854.24	*D*_x_ = 1.275 Mg m^−^^3^
Triclinic, *P*1	Melting point: 331 K
*a* = 7.484 (1) Å	Mo *K*α radiation, λ = 0.71073 Å
*b* = 8.587 (1) Å	Cell parameters from 4916 reflections
*c* = 18.523 (2) Å	θ = 2.5–23.6°
α = 83.782 (4)°	µ = 0.61 mm^−^^1^
β = 81.868 (4)°	*T* = 123 K
γ = 71.189 (4)°	Leaf, blue
*V* = 1112.9 (2) Å^3^	0.17 × 0.10 × 0.05 mm
*Z* = 1	

### Data collection

**Table:**

Bruker Kappa APEXII CCD diffractometer	4509 reflections with *I* > 2σ(*I*)
Radiation source: sealed tube	*R*_int_ = 0.084
φ and ω scans	θ_max_ = 30.5°, θ_min_ = 2.7°
Absorption correction: multi-scan (*SADABS*; Bruker, 2017)	*h* = −10→10
	*k* = −12→12
35032 measured reflections	*l* = −26→26
6802 independent reflections	

### Refinement

**Table:**

Refinement on *F*^2^	Primary atom site location: structure-invariant direct methods
Least-squares matrix: full	Secondary atom site location: difference Fourier map
*R*[*F*^2^ > 2σ(*F*^2^)] = 0.049	Hydrogen site location: inferred from neighbouring sites
*wR*(*F*^2^) = 0.106	H-atom parameters constrained
*S* = 1.04	*w* = 1/[σ^2^(*F*_o_^2^) + (0.0377*P*)^2^ + 0.289*P*] where *P* = (*F*_o_^2^ + 2*F*_c_^2^)/3
6802 reflections	(Δ/σ)_max_ < 0.001
241 parameters	Δρ_max_ = 0.53 e Å^−^^3^
0 restraints	Δρ_min_ = −0.65 e Å^−^^3^

### Special details

**Table:**

Geometry. All esds (except the esd in the dihedral angle between two l.s. planes) are estimated using the full covariance matrix. The cell esds are taken into account individually in the estimation of esds in distances, angles and torsion angles; correlations between esds in cell parameters are only used when they are defined by crystal symmetry. An approximate (isotropic) treatment of cell esds is used for estimating esds involving l.s. planes.

### Fractional atomic coordinates and isotropic or equivalent isotropic displacement parameters (Å^2^)

**Table:**

	*x*	*y*	*z*	*U*_iso_*/*U*_eq_	
Co1	0.0000	1.0000	0.0000	0.01303 (10)	
C1	0.3937 (3)	0.7311 (2)	−0.0519 (1)	0.0148 (4)	
N1	0.2432 (2)	0.8063 (2)	−0.0267 (1)	0.0184 (4)	
S1	0.60623 (7)	0.63024 (7)	−0.08753 (3)	0.0220 (1)	
C2	0.1513 (3)	1.0644 (3)	0.1446 (1)	0.0175 (4)	
N2	0.1189 (2)	1.0506 (2)	0.0868 (1)	0.0191 (4)	
S2	0.19619 (9)	1.07863 (8)	0.22694 (3)	0.0323 (2)	
N3	−0.1489 (2)	0.8338 (2)	0.07881 (9)	0.0134 (3)	
C3	−0.1506 (3)	0.8560 (3)	0.1567 (1)	0.0236 (5)	
H3A	−0.2029	0.9748	0.1654	0.028*	
H3B	−0.0188	0.8157	0.1698	0.028*	
C4	−0.2714 (3)	0.7615 (3)	0.2057 (1)	0.0216 (5)	
H4A	−0.2066	0.7072	0.2491	0.026*	
H4B	−0.3964	0.8392	0.2229	0.026*	
N4	−0.2984 (2)	0.6345 (2)	0.16244 (9)	0.0146 (3)	
C5	−0.0444 (3)	0.6600 (3)	0.0655 (1)	0.0242 (5)	
H5A	0.0933	0.6407	0.0656	0.029*	
H5B	−0.0650	0.6366	0.0167	0.029*	
C6	−0.1092 (3)	0.5418 (3)	0.1239 (1)	0.0226 (5)	
H6A	−0.1203	0.4474	0.1006	0.027*	
H6B	−0.0147	0.4986	0.1594	0.027*	
C7	−0.3483 (3)	0.8618 (2)	0.0661 (1)	0.0167 (4)	
H7A	−0.3557	0.8665	0.0129	0.020*	
H7B	−0.4288	0.9693	0.0843	0.020*	
C8	−0.4249 (3)	0.7243 (3)	0.1047 (1)	0.0182 (4)	
H8A	−0.5568	0.7725	0.1275	0.022*	
H8B	−0.4251	0.6468	0.0689	0.022*	
C9	−0.3746 (3)	0.5126 (3)	0.2108 (1)	0.0193 (4)	
H9A	−0.2860	0.4609	0.2478	0.023*	
H9B	−0.3746	0.4244	0.1805	0.023*	
C10	−0.5729 (3)	0.5808 (3)	0.2504 (1)	0.0222 (5)	
H10A	−0.6664	0.6223	0.2144	0.027*	
H10B	−0.5786	0.6739	0.2788	0.027*	
C11	−0.6218 (3)	0.4455 (3)	0.3016 (1)	0.0225 (5)	
H11A	−0.5944	0.3462	0.2741	0.027*	
H11B	−0.5386	0.4162	0.3413	0.027*	
C12	−0.8274 (3)	0.4935 (3)	0.3352 (1)	0.0239 (5)	
H12A	−0.9105	0.5135	0.2959	0.029*	
H12B	−0.8581	0.5978	0.3594	0.029*	
C13	−0.8697 (3)	0.3628 (3)	0.3908 (1)	0.0222 (5)	
H13A	−0.7909	0.3478	0.4312	0.027*	
H13B	−0.8301	0.2570	0.3672	0.027*	
C14	−1.0763 (3)	0.3994 (3)	0.4232 (1)	0.0236 (5)	
H14A	−1.1560	0.4109	0.3833	0.028*	
H14B	−1.1179	0.5056	0.4465	0.028*	
C15	−1.1072 (3)	0.2649 (3)	0.4796 (1)	0.0231 (5)	
H15A	−1.0546	0.1576	0.4571	0.028*	
H15B	−1.0344	0.2598	0.5209	0.028*	
C16	−1.3133 (3)	0.2878 (3)	0.5096 (1)	0.0238 (5)	
H16A	−1.3616	0.3877	0.5376	0.029*	
H16B	−1.3895	0.3062	0.4681	0.029*	
C17	−1.3421 (3)	0.1423 (3)	0.5586 (1)	0.0230 (5)	
H17A	−1.2724	0.1286	0.6017	0.028*	
H17B	−1.2857	0.0412	0.5316	0.028*	
C18	−1.5491 (3)	0.1587 (3)	0.5851 (1)	0.0232 (5)	
H18A	−1.6022	0.2531	0.6164	0.028*	
H18B	−1.6217	0.1832	0.5423	0.028*	
C19	−1.5760 (3)	0.0051 (3)	0.6280 (1)	0.0250 (5)	
H19A	−1.5108	−0.0148	0.6726	0.030*	
H19B	−1.5146	−0.0908	0.5980	0.030*	
C20	−1.7843 (3)	0.0161 (3)	0.6502 (1)	0.0291 (5)	
H20A	−1.7914	−0.0865	0.6776	0.044*	
H20B	−1.8494	0.0328	0.6063	0.044*	
H20C	−1.8457	0.1089	0.6810	0.044*	

### Atomic displacement parameters (Å^2^)

**Table:**

	*U*^11^	*U*^22^	*U*^33^	*U*^12^	*U*^13^	*U*^23^
Co1	0.0092 (2)	0.0119 (2)	0.0172 (2)	−0.0013 (2)	−0.0026 (2)	−0.0027 (2)
C1	0.020 (1)	0.011 (1)	0.017 (1)	−0.0075 (8)	−0.0064 (8)	0.0014 (8)
N1	0.0135 (8)	0.0145 (9)	0.025 (1)	−0.0016 (7)	−0.0012 (7)	−0.0015 (7)
S1	0.0133 (3)	0.0218 (3)	0.0299 (3)	−0.0033 (2)	0.0022 (2)	−0.0099 (2)
C2	0.018 (1)	0.017 (1)	0.020 (1)	−0.0100 (8)	−0.0006 (8)	−0.0008 (8)
N2	0.0179 (9)	0.021 (1)	0.021 (1)	−0.0089 (7)	−0.0026 (7)	−0.0018 (7)
S2	0.0414 (4)	0.0447 (4)	0.0188 (3)	−0.0227 (3)	−0.0072 (3)	−0.0023 (3)
N3	0.0098 (7)	0.0124 (8)	0.0172 (9)	−0.0015 (6)	−0.0027 (6)	−0.0020 (7)
C3	0.029 (1)	0.031 (1)	0.018 (1)	−0.020 (1)	−0.0030 (9)	−0.0015 (9)
C4	0.026 (1)	0.026 (1)	0.019 (1)	−0.014 (1)	−0.0029 (9)	−0.0047 (9)
N4	0.0134 (8)	0.0138 (9)	0.0166 (9)	−0.0050 (7)	−0.0017 (7)	0.0007 (7)
C5	0.020 (1)	0.014 (1)	0.031 (1)	0.0015 (9)	0.0048 (9)	0.0006 (9)
C6	0.016 (1)	0.017 (1)	0.028 (1)	0.0009 (8)	0.0028 (9)	0.0025 (9)
C7	0.0131 (9)	0.016 (1)	0.021 (1)	−0.0047 (8)	−0.0064 (8)	0.0031 (8)
C8	0.019 (1)	0.020 (1)	0.018 (1)	−0.0089 (9)	−0.0081 (8)	0.0051 (8)
C9	0.022 (1)	0.020 (1)	0.018 (1)	−0.0096 (9)	−0.0036 (8)	0.0038 (8)
C10	0.022 (1)	0.021 (1)	0.024 (1)	−0.0084 (9)	−0.0003 (9)	0.0009 (9)
C11	0.024 (1)	0.023 (1)	0.022 (1)	−0.0095 (9)	−0.0020 (9)	0.0025 (9)
C12	0.022 (1)	0.023 (1)	0.025 (1)	−0.0073 (9)	−0.0015 (9)	0.0034 (9)
C13	0.022 (1)	0.023 (1)	0.022 (1)	−0.0078 (9)	−0.0005 (9)	0.0004 (9)
C14	0.020 (1)	0.024 (1)	0.027 (1)	−0.0075 (9)	−0.0021 (9)	0.0009 (9)
C15	0.018 (1)	0.022 (1)	0.028 (1)	−0.0050 (9)	−0.0009 (9)	0.0001 (9)
C16	0.020 (1)	0.020 (1)	0.029 (1)	−0.0050 (9)	−0.0018 (9)	0.0017 (9)
C17	0.020 (1)	0.021 (1)	0.025 (1)	−0.0044 (9)	0.0004 (9)	0.0026 (9)
C18	0.019 (1)	0.021 (1)	0.028 (1)	−0.0049 (9)	−0.0007 (9)	−0.0013 (9)
C19	0.025 (1)	0.026 (1)	0.024 (1)	−0.010 (1)	−0.0016 (9)	−0.001 (1)
C20	0.028 (1)	0.034 (1)	0.029 (1)	−0.015 (1)	0.000 (1)	−0.003 (1)

### Geometric parameters (Å, º)

**Table:**

Co1—N1^i^	2.072 (2)	C9—H9B	0.9900
Co1—N1	2.073 (2)	C10—C11	1.523 (3)
Co1—N2	2.090 (2)	C10—H10A	0.9900
Co1—N2^i^	2.090 (2)	C10—H10B	0.9900
Co1—N3	2.350 (2)	C11—C12	1.517 (3)
Co1—N3^i^	2.350 (2)	C11—H11A	0.9900
C1—N1	1.162 (3)	C11—H11B	0.9900
C1—S1	1.629 (2)	C12—C13	1.518 (3)
C2—N2	1.158 (3)	C12—H12A	0.9900
C2—S2	1.633 (2)	C12—H12B	0.9900
N3—C3	1.474 (3)	C13—C14	1.521 (3)
N3—C5	1.475 (2)	C13—H13A	0.9900
N3—C7	1.482 (2)	C13—H13B	0.9900
C3—C4	1.543 (3)	C14—C15	1.524 (3)
C3—H3A	0.9900	C14—H14A	0.9900
C3—H3B	0.9900	C14—H14B	0.9900
C4—N4	1.500 (3)	C15—C16	1.521 (3)
C4—H4A	0.9900	C15—H15A	0.9900
C4—H4B	0.9900	C15—H15B	0.9900
N4—C6	1.503 (2)	C16—C17	1.518 (3)
N4—C9	1.504 (2)	C16—H16A	0.9900
N4—C8	1.507 (2)	C16—H16B	0.9900
C5—C6	1.538 (3)	C17—C18	1.523 (3)
C5—H5A	0.9900	C17—H17A	0.9900
C5—H5B	0.9900	C17—H17B	0.9900
C6—H6A	0.9900	C18—C19	1.520 (3)
C6—H6B	0.9900	C18—H18A	0.9900
C7—C8	1.540 (3)	C18—H18B	0.9900
C7—H7A	0.9900	C19—C20	1.530 (3)
C7—H7B	0.9900	C19—H19A	0.9900
C8—H8A	0.9900	C19—H19B	0.9900
C8—H8B	0.9900	C20—H20A	0.9800
C9—C10	1.519 (3)	C20—H20B	0.9800
C9—H9A	0.9900	C20—H20C	0.9800
			
N1^i^—Co1—N1	180.0	N4—C9—H9B	108.2
N1^i^—Co1—N2	88.95 (7)	C10—C9—H9B	108.2
N1—Co1—N2	91.05 (7)	H9A—C9—H9B	107.4
N1^i^—Co1—N2^i^	91.05 (7)	C9—C10—C11	109.6 (2)
N1—Co1—N2^i^	88.95 (7)	C9—C10—H10A	109.7
N2—Co1—N2^i^	180.0	C11—C10—H10A	109.7
N1^i^—Co1—N3	85.89 (6)	C9—C10—H10B	109.7
N1—Co1—N3	94.11 (6)	C11—C10—H10B	109.7
N2—Co1—N3	90.94 (6)	H10A—C10—H10B	108.2
N2^i^—Co1—N3	89.06 (6)	C12—C11—C10	113.7 (2)
N1^i^—Co1—N3^i^	94.11 (6)	C12—C11—H11A	108.8
N1—Co1—N3^i^	85.89 (6)	C10—C11—H11A	108.8
N2—Co1—N3^i^	89.06 (6)	C12—C11—H11B	108.8
N2^i^—Co1—N3^i^	90.94 (6)	C10—C11—H11B	108.8
N3—Co1—N3^i^	180.0	H11A—C11—H11B	107.7
N1—C1—S1	178.5 (2)	C11—C12—C13	113.0 (2)
C1—N1—Co1	162.0 (2)	C11—C12—H12A	109.0
N2—C2—S2	178.3 (2)	C13—C12—H12A	109.0
C2—N2—Co1	163.3 (2)	C11—C12—H12B	109.0
C3—N3—C5	108.1 (2)	C13—C12—H12B	109.0
C3—N3—C7	107.1 (2)	H12A—C12—H12B	107.8
C5—N3—C7	106.8 (2)	C12—C13—C14	115.4 (2)
C3—N3—Co1	113.2 (1)	C12—C13—H13A	108.4
C5—N3—Co1	108.1 (1)	C14—C13—H13A	108.4
C7—N3—Co1	113.3 (1)	C12—C13—H13B	108.4
N3—C3—C4	111.1 (2)	C14—C13—H13B	108.4
N3—C3—H3A	109.4	H13A—C13—H13B	107.5
C4—C3—H3A	109.4	C13—C14—C15	112.5 (2)
N3—C3—H3B	109.4	C13—C14—H14A	109.1
C4—C3—H3B	109.4	C15—C14—H14A	109.1
H3A—C3—H3B	108.0	C13—C14—H14B	109.1
N4—C4—C3	108.9 (2)	C15—C14—H14B	109.1
N4—C4—H4A	109.9	H14A—C14—H14B	107.8
C3—C4—H4A	109.9	C16—C15—C14	114.9 (2)
N4—C4—H4B	109.9	C16—C15—H15A	108.5
C3—C4—H4B	109.9	C14—C15—H15A	108.5
H4A—C4—H4B	108.3	C16—C15—H15B	108.5
C4—N4—C6	108.7 (2)	C14—C15—H15B	108.5
C4—N4—C9	111.6 (2)	H15A—C15—H15B	107.5
C6—N4—C9	108.2 (2)	C17—C16—C15	113.7 (2)
C4—N4—C8	107.7 (2)	C17—C16—H16A	108.8
C6—N4—C8	107.5 (2)	C15—C16—H16A	108.8
C9—N4—C8	113.0 (2)	C17—C16—H16B	108.8
N3—C5—C6	111.6 (2)	C15—C16—H16B	108.8
N3—C5—H5A	109.3	H16A—C16—H16B	107.7
C6—C5—H5A	109.3	C16—C17—C18	114.3 (2)
N3—C5—H5B	109.3	C16—C17—H17A	108.7
C6—C5—H5B	109.3	C18—C17—H17A	108.7
H5A—C5—H5B	108.0	C16—C17—H17B	108.7
N4—C6—C5	108.7 (2)	C18—C17—H17B	108.7
N4—C6—H6A	110.0	H17A—C17—H17B	107.6
C5—C6—H6A	110.0	C19—C18—C17	113.4 (2)
N4—C6—H6B	110.0	C19—C18—H18A	108.9
C5—C6—H6B	110.0	C17—C18—H18A	108.9
H6A—C6—H6B	108.3	C19—C18—H18B	108.9
N3—C7—C8	111.7 (2)	C17—C18—H18B	108.9
N3—C7—H7A	109.3	H18A—C18—H18B	107.7
C8—C7—H7A	109.3	C18—C19—C20	113.7 (2)
N3—C7—H7B	109.3	C18—C19—H19A	108.8
C8—C7—H7B	109.3	C20—C19—H19A	108.8
H7A—C7—H7B	107.9	C18—C19—H19B	108.8
N4—C8—C7	108.3 (2)	C20—C19—H19B	108.8
N4—C8—H8A	110.0	H19A—C19—H19B	107.7
C7—C8—H8A	110.0	C19—C20—H20A	109.5
N4—C8—H8B	110.0	C19—C20—H20B	109.5
C7—C8—H8B	110.0	H20A—C20—H20B	109.5
H8A—C8—H8B	108.4	C19—C20—H20C	109.5
N4—C9—C10	116.2 (2)	H20A—C20—H20C	109.5
N4—C9—H9A	108.2	H20B—C20—H20C	109.5
C10—C9—H9A	108.2		
			
C5—N3—C3—C4	−67.5 (2)	C4—N4—C8—C7	48.3 (2)
C7—N3—C3—C4	47.2 (2)	C6—N4—C8—C7	−68.7 (2)
Co1—N3—C3—C4	172.8 (1)	C9—N4—C8—C7	172.1 (2)
N3—C3—C4—N4	18.0 (2)	N3—C7—C8—N4	18.0 (2)
C3—C4—N4—C6	47.5 (2)	C4—N4—C9—C10	64.9 (2)
C3—C4—N4—C9	166.7 (2)	C6—N4—C9—C10	−175.6 (2)
C3—C4—N4—C8	−68.7 (2)	C8—N4—C9—C10	−56.7 (2)
C3—N3—C5—C6	46.8 (2)	N4—C9—C10—C11	−175.2 (2)
C7—N3—C5—C6	−68.0 (2)	C9—C10—C11—C12	−171.3 (2)
Co1—N3—C5—C6	169.7 (1)	C10—C11—C12—C13	−175.3 (2)
C4—N4—C6—C5	−67.5 (2)	C11—C12—C13—C14	−176.5 (2)
C9—N4—C6—C5	171.1 (2)	C12—C13—C14—C15	−178.7 (2)
C8—N4—C6—C5	48.9 (2)	C13—C14—C15—C16	−175.4 (2)
N3—C5—C6—N4	17.8 (3)	C14—C15—C16—C17	173.1 (2)
C3—N3—C7—C8	−68.4 (2)	C15—C16—C17—C18	−176.3 (2)
C5—N3—C7—C8	47.2 (2)	C16—C17—C18—C19	174.4 (2)
Co1—N3—C7—C8	166.1 (1)	C17—C18—C19—C20	−176.0 (2)

Symmetry code: (i) −*x*, −*y*+2, −*z*.

### Hydrogen-bond geometry (Å, º)

**Table:**

*D*—H···*A*	*D*—H	H···*A*	*D*···*A*	*D*—H···*A*
C5—H5*A*···N1	0.99	2.48	3.060 (3)	117
C5—H5*A*···S1^ii^	0.99	2.70	3.467 (2)	134
C7—H7*A*···S1^iii^	0.99	3.00	3.755 (2)	134
C7—H7*A*···N2^i^	0.99	2.58	3.244 (3)	124
C8—H8*B*···S1^iii^	0.99	2.89	3.695 (2)	139
C9—H9*B*···S1^iv^	0.99	2.78	3.580 (2)	138

Symmetry codes: (i) −*x*, −*y*+2, −*z*; (ii) −*x*+1, −*y*+1, −*z*; (iii) *x*−1, *y*, *z*; (iv) −*x*, −*y*+1, −*z*.
